# Linking Circadian Rhythms to Gut‐Brain Axis Lipid Metabolism Associated With Endoplasmic Reticulum Stress in Alzheimer's Disease

**DOI:** 10.1111/cns.70329

**Published:** 2025-03-09

**Authors:** Jianhui Su, Lanyang Zhao, Runze Fu, Zhe Tang

**Affiliations:** ^1^ School of Marine and Bioengineering Yancheng Institute of Technology Yancheng Jiangsu China; ^2^ School of Pharmacy Nanjing University of Chinese Medicine Nanjing China; ^3^ School of Chemistry & Chemical Engineering Yancheng Institute of Technology Yancheng Jiangsu China

**Keywords:** AD, circadian rhythm, ERS, inflammation, intestinal mucosal barrier, lipid homeostasis

## Abstract

**Background:**

Alzheimer's disease (AD) is characterized by a decline in cognitive, learning, and memory abilities. Neuroinflammation is associated with the spread of tau tangles in the neocortex of AD, leading to cognitive impairment. Therefore, clarifying the pathogenesis of Neuroinflammation and finding effective treatments are the crucial issues for the clinical management of AD.

**Method:**

We systematically review the latest research on the pathogenesis and therapeutic strategies of AD in PubMed, Web of Science, and Elsevier SD.

**Result:**

In this review, the mechanism of the effect of gut‐brain axis lipid metabolism mediated by circadian rhythm on AD was discussed, and we also analysed the effects of inflammation and endoplasmic reticulum stress (ERS) induced by lipid abnormalities on intestinal mucosal barrier and neurodegeneration; furthermore, the importance of lipid homeostasis (phospholipids, fatty acids, sterol) in maintaining the functions of endoplasmic reticulum was emphasized. Meanwhile, as lipid composition affects protein conformation, the membrane phospholipids surrounding sarcoplasmic reticulum Ca^2+^‐ATPase (SERCA) that influence SERCA to release Ca^2+^ mediating inflammation were also reviewed.

**Conclusion:**

We interpreted the mechanism of action between lipid microdomains and ER membrane proteins, reviewed the role of the new pathway of circadian rhythm, lipid metabolism, intestinal mucosa, and brain signaling in the pathogenesis of AD, and proposed strategies to prevent AD by changing the dietary lipid measures.

## Graphical abstract

The effect of gut‐brain axis lipid abnormalities metabolism mediated by circadian rhythm disruption on AD were discussed. The membrane lipids abnormalities surrounding sarcoplasmic reticulum Ca^2+^ ‐ATPase (SERCA) influencing SERCA to release Ca^2+^ mediate ERS and inflammation, which contribute to neuron damage Aβ accumulation in AD. In addition, Intestinal mucosal ERS leads to increased permeability, and accelerate AD progression by the increase of Lipopolysaccharide and proinflammatory factor.

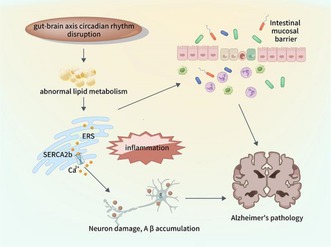

## 1 Introduction

AD is a neurodegenerative disorder characterized by cognitive impairment. According to the World Health Organization, approximately 115 million individuals will be affected by AD if effective therapeutic interventions are not implemented by 2050 [1]. The presence of amyloid plaques in the brains of many elderly individuals does not necessarily lead to the development of AD. The interaction between neuroinflammation and amyloid‐beta (Aβ) lesions promotes the propagation of tau tangles, which drive the progression of AD [2]. Therefore, precise regulation of neuroinflammation is crucial to prevent AD. Lipopolysaccharide (LPS) produced by an imbalance in the intestinal microbiota permeates the brain through the intestinal mucosal barrier and modulates microglial phagocytic activity towards Aβ in the brain. This leads to impaired clearance of Aβ deposition in the brain, while simultaneously triggering excessive activation of microglia and subsequent expression of pro‐inflammatory factors [3]. Lipidomics, particularly the ERS mediated by gut‐brain axis lipid composition, has emerged as a prominent area of investigation. A growing body of experimental evidence suggests an interaction between the circadian clock and abnormal lipid metabolism or obesity [4]. The lipid composition of the intestinal mucosa mediates ERS, regulates the expression of pro‐inflammatory factors, and influences the integrity and permeability of the intestinal mucosa [5]. The circadian rhythm regulates the expression of tight junction proteins, including occludin and claudin1, leading to improved barrier function against LPS‐induced permeability [6]. Thus, circadian rhythm mediates gut‐brain axis lipid metabolism affecting the intestinal mucosal barrier and cognitive function.

In this paper, we present a comprehensive review of the recent advancements in the field of associations between dietary patterns and AD, along with their underlying mechanisms. Investigating the interplay between lipid microdomains and ER membrane proteins is important in elucidating the regulatory mechanism through which circadian rhythms, lipid metabolism, and mucosal‐brain signals regulate nervous system development, behavior, memory, and cognition.

## 2 Circadian Rhythms Affect Cognitive Function

### 2.1 The Gut‐Brain Axis Communicates Bidirectionally Through the Intestinal Mucosal Barrier

Studies on the microbial‐gut‐brain axis suggest that gut microbes have the potential to influence the nervous system through various mechanisms, including direct or indirect regulation of neurotransmitter synthesis and modulation of neuronal and glial cell survival and function, as well as alteration of immune responses within the nervous system [7]. The human intestinal mucosal barrier consists of mechanical, chemical, immune, and biological components [8]. The alteration in intestinal mucosal permeability is closely associated with injury to intestinal epithelial cells and the downregulation of tight junction protein expression [9]. Intestinal flora and endotoxins enter the blood through mucous membranes and spread to various tissues and organs in the body, leading to systemic chronic inflammation [10]. This article reviews the effect of intestinal epithelial integrity on neuroinflammation.

### 2.2 Circadian Rhythm Mediates Gut‐Brain Axis Lipid Metabolism Affecting Intestinal Mucosal Barrier and Cognitive Function

#### 2.2.1 Clock‐BMAL1

**FIGURE 1:**
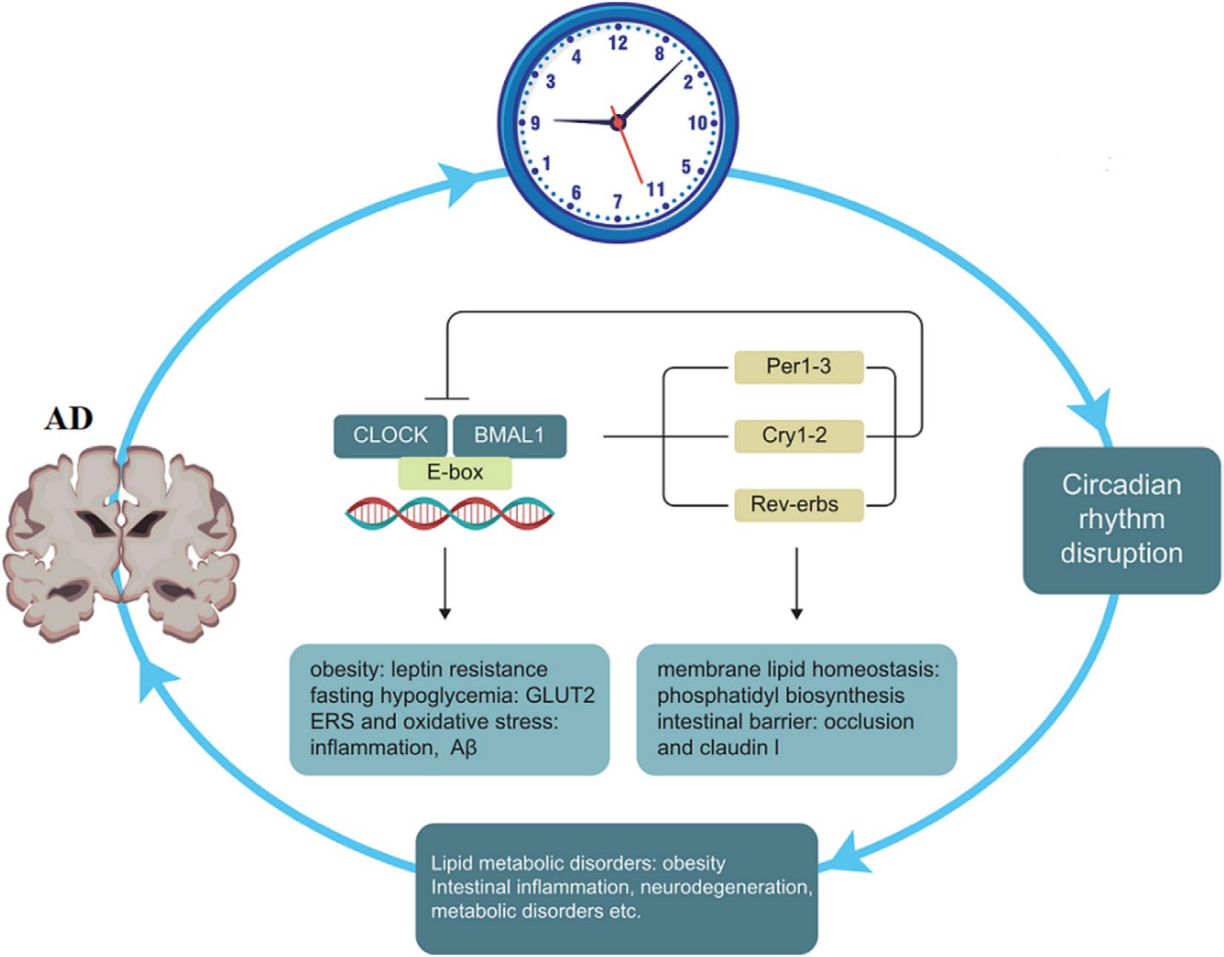
Disruption of the circadian clock induces neurodegenerative changes in the brain through various mechanisms. Transcriptional regulation of CLOCK‐BMAL1 represents one potential mechanism for regulating obesity, oxidative stress, and ERS. Furthermore, REV‐ERBα along with the circadian cycle, plays a critical role in regulating disorders related to lipid metabolism and neuroinflammation.

The suprachiasmatic nucleus (SCN) within the hypothalamus serves as the primary coordinator of most circadian rhythms. Light stimuli prompt the SCN to synchronize with the ambient light–dark cycle. Additionally, the expression of circadian clock genes has been detected in nearly all cells outside the SCN, and these rhythmic expressions persist even in vitro. Circadian rhythms refer to the rhythmic changes in a mammal's 24‐h cycle, including sleep patterns, dietary habits, hormone secretion, and body temperature fluctuations [11, 12, 13]. Disruptions in circadian rhythm cause significant risks for aberrant sleep–wake cycles, diabetes, obesity, cancer, and other diseases [14]. Biological clocks are regulated by core clock genes, such as Bmal1, circadian locomotor output cycles kaput (CLOCK), cyclic circadian rhythm proteins (Pers; Per1, Per2 and Per3), and cryptochrome (Crys; Cry1 and Cry2). These fundamental clock genes are widely conserved across plants, animals, and microorganisms [15]. The Clock protein interacts with Bmal1 to form a heterodimer known as the Clock‐Bmal1 complex. This complex enhances the expression of Per 1/2/3 and Cry 1/2 in the cytoplasm. Subsequently, this complex translocates into the nucleus where it functions as a transcriptional inhibitor to suppress Clock‐Bmal1‐mediated gene transcription, thereby establishing a negative feedback loop (Figure 1). Additionally, the Clock‐Bmal1 heterodimer positively regulates nuclear hormone receptors (Rev‐erbs; Rev‐erα/β), which act as transcriptional repressors. These receptors enter the nucleus and competitively bind to the Bmal1 promoter element to inhibit Bmal1 expression, thus forming another negative feedback loop.

#### 2.2.2 Circadian Rhythms Affect Lipid Metabolism

It has been reported that disruption of circadian regulation leads to increased levels of various phospholipids in plasma, consequently disturbing membrane lipid homeostasis [16, 17]. Rev‐erbα controls the rhythmic expression of genes involved in fatty acid biosynthesis and phosphatidyl biosynthesis, such as choline kinaseα(Chkα) (Figure 1) [18]. Circadian misalignment is commonly observed in individuals with obesity who tend to have nocturnal overeating [19]. Disruptions in circadian eating behavior are induced by mutations in the Bmal1 and Clock genes, ultimately leading to overall hyperactivity in diet‐induced obese mice [20, 21]. Reduced Clock expression diminishes leptin sensitivity in obese mice; alterations are observed in Clock‐regulated gene expression such as glucose transporter 2 (GLUT2) and glucose isomer genes, contributing to fasting hypoglycemia (Figure 1) [22]. Knockout of the Bmal1 gene leads to a reduction in lipid gene expression, such as stearoyl‐CoA desaturase, and decreases synthesis of long‐chain polyunsaturated fatty acids (PUFA) in adipose tissue [23]. PUFA display a circadian rhythm, with peak levels observed 9 h after exposure to light; diurnal oscillations are evident in nuclear lipids (34%) and mitochondrial lipids (31%), primarily composed of phospholipids [24].

#### 2.2.3 Circadian Rhythms Affect Intestinal Mucosal Barrier and Cognitive Function

AD is characterized by tau aggregation and is associated with disrupted circadian rhythms and suppression of clock gene expression. It has been reported that the circadian clock protein Rev‐erbα is a key regulator between the biological clock, glial activation, and neuroinflammation, and loss of rev‐erbα triggers activation of microglia, increases nuclear factor κB(NF‐κB) signaling in microglia, leading to neuronal damage [25]. Lee found in male mice that Rev‐erbα is involved in lipid metabolism and macrophage function, and that Rev‐erbα deletion causes abnormal lipid metabolism in microglia, induces lipid droplet accumulation, and leads to lipid droplet accumulation specifically in male microglia, which damages tau phagocytosis in microglia and exacerbates neuroinflammation [26].

Furthermore, in intestinal immune cells, the biological clock collaborates with the microbiome to regulate the circadian rhythm of dietary lipid absorption, which reaches its maximum during nighttime [27]. The transcription suppressor nuclear factor interleukin 3 (NFiL3), a leucine zipper transcription factor, is expressed in various immune cells and regulates lipid absorption and export in small intestinal epithelial cells. The expression of Nfil3 is regulated by the Clock transcription suppressor REV‐ERBα, resulting in a rhythmic diurnal pattern of Nfil3 expression [28].

The intestinal barrier plays a critical role in maintaining intestinal homeostasis. Changes in tight junctions or reduced mucin protection can impact the permeability of the intestinal barrier, which is influenced by circadian rhythm. CLOCK‐BMAL1 binding to the E‐box on the promoter region positively regulates nuclear hormone receptors Rev‐erbα, which induce occludin and claudin 1 expression in mouse and human intestinal epithelial cells [10, 29]. The expressions of claudin 1 and occludin were regulated by the circadian clock, with both mRNA and protein levels reaching their lowest point at night and peaking during the day [29]. Tan et al. investigated the mechanism of acrylamide‐induced neurotoxicity related to the circadian clock in mice brains. Their study revealed that an acrylamide‐containing diet significantly decreased occludin protein levels and exacerbated cognitive dysfunction and spatial memory impairment during the night phase. This effect suggested that circadian rhythm disruptor compromised intestinal barrier integrity and increased circulating levels of LPS, Interleukin1β(IL‐1β), and tumor necrosis factorα(TNF‐α) [30]. In another study, disruption of the circadian rhythm was shown to modulate the composition of gut microbiota, impair intestinal barrier integrity, and induce both peripheral and central inflammation as well as cognitive impairment in mice. These alterations were accompanied by AD‐like tau hyperphosphorylation, defects in neurogenesis, and a loss of synaptic proteins in the hippocampus in mice with disrupted circadian rhythms [31]. Additionally, Song et al. reported that oolong tea polyphenols alleviated intestinal barrier damage and reduced LPS transport to the serum by regulating gut microbiota composition, thereby mitigating circadian rhythm disruption‐induced cognitive decline [32]. Therefore, maintaining a regular circadian rhythm enhances intestinal barrier integrity and effectively suppresses pathological changes and dysfunctions.

### 2.3 Circadian Rhythms Mediate Stress (ERS, Oxidative Stress) Effects on Cognitive Function

#### 2.3.1 Circadian Rhythms Mediate the Effects of ERS on Cognitive Function

In certain physiological or pathological conditions, the demand for protein biosynthesis can increase dramatically, surpassing the folding capacity of the endoplasmic reticulum (ER), leading to the accumulation of partially folded, misfolded, or unfolded proteins, a condition known as ERS. The buildup of these aberrant proteins within the ER lumen triggers the activation of the unfolded protein response (UPR) signaling pathway. Mild ER stress elicits an “adaptive or protective UPR” that prevents excessive accumulation of misfolded/unfolded proteins in the ER. However, excessive or prolonged ERS can lead to sustained activation of the UPR, referred to as “maladaptive or terminal UPR”, which exacerbates neuroinflammation and triggers cell death [33]. In recent years, a large amount of evidence has shown that ERS is important in the development of AD [34], and that protein degradation regulated by UPR affects the excessive phosphoric acid of neurofibrillary tangles. Circadian rhythm disruptions can occur at an early stage in specific neurodegenerative conditions, the misalignment of circadian rhythms cause ERS [35]. Melatonin is known for its role in regulating circadian rhythms, sleep, and aging. The deficiency of melatonin in cerebrospinal fluid promotes the progression of cognitive impairment, and melatonin supplementation can effectively slow down the process of degeneration of hippocampal neurons and improve cognitive ability. Melatonin protects neurons by alleviating Ca^2+^ overload and ERS, regulating intracellular Ca^2+^ levels and cell homeostasis [36]. Kainic Acid induced ERS, affecting CDK5/GSK‐3β and tau phosphorylation. Melatonin plays a role in Ka‐induced neuronal degeneration and tau hyperphosphorylation by relieving ERS, further emphasizing the protective role of melatonin in Ka‐induced neurons. Furthermore, in rats exposed to long‐term light, circadian rhythms were disrupted, ERS‐related proteins were increased, and melatonin supplementation prevented molecular damage [37]. Therefore, the interaction between circadian rhythm and ERS is a target for melatonin in therapy in neurodegenerative diseases.

#### 2.3.2 Circadian Rhythms Mediate Effects of Oxidative Stress on Cognitive Function

Meanwhile, disruption of the circadian rhythm is associated with increased neuronal oxidative stress, resulting in lipid peroxidation as well as protein and nucleic acid oxidation, which are implicated in the early pathogenesis of AD [34, 38]. Bmal1 coordinates the transcription of genes related to REDOX processes in the brain, including quinone oxidoreductase antibody and acetaldehyde dehydrogenase. Moreover, reduced Bmal1 expression exacerbates neuronal cell death induced by oxidative stress both in vivo and in vitro [39]. Neuroinflammation spreads through the activation of astrocytes and microglia, contributing to neurodegeneration [39]. The inflammatory response exhibited by microglia indicates that inflammation in the hippocampus or cortex can directly hinder Bmal1 expression in peripheral neurons and glial cells, thus impairing the expression of Bmal1‐mediated oxidative stress genes and leading to neurodegeneration in these cells. Rev‐erbα‐Bmal1 regulates pro‐inflammatory cytokine production in macrophages [40]. Additionally, inflammation disrupts the circadian clock as LPS inhibits Bmal1 and REV‐ERBα levels in macrophages.

## 3 Lipid Metabolism Mediates Serca2b Activity, Influences ERS, and Mediates Inflammatory Expression

### 3.1 Lipids Affect ERS

**FIGURE 2:**
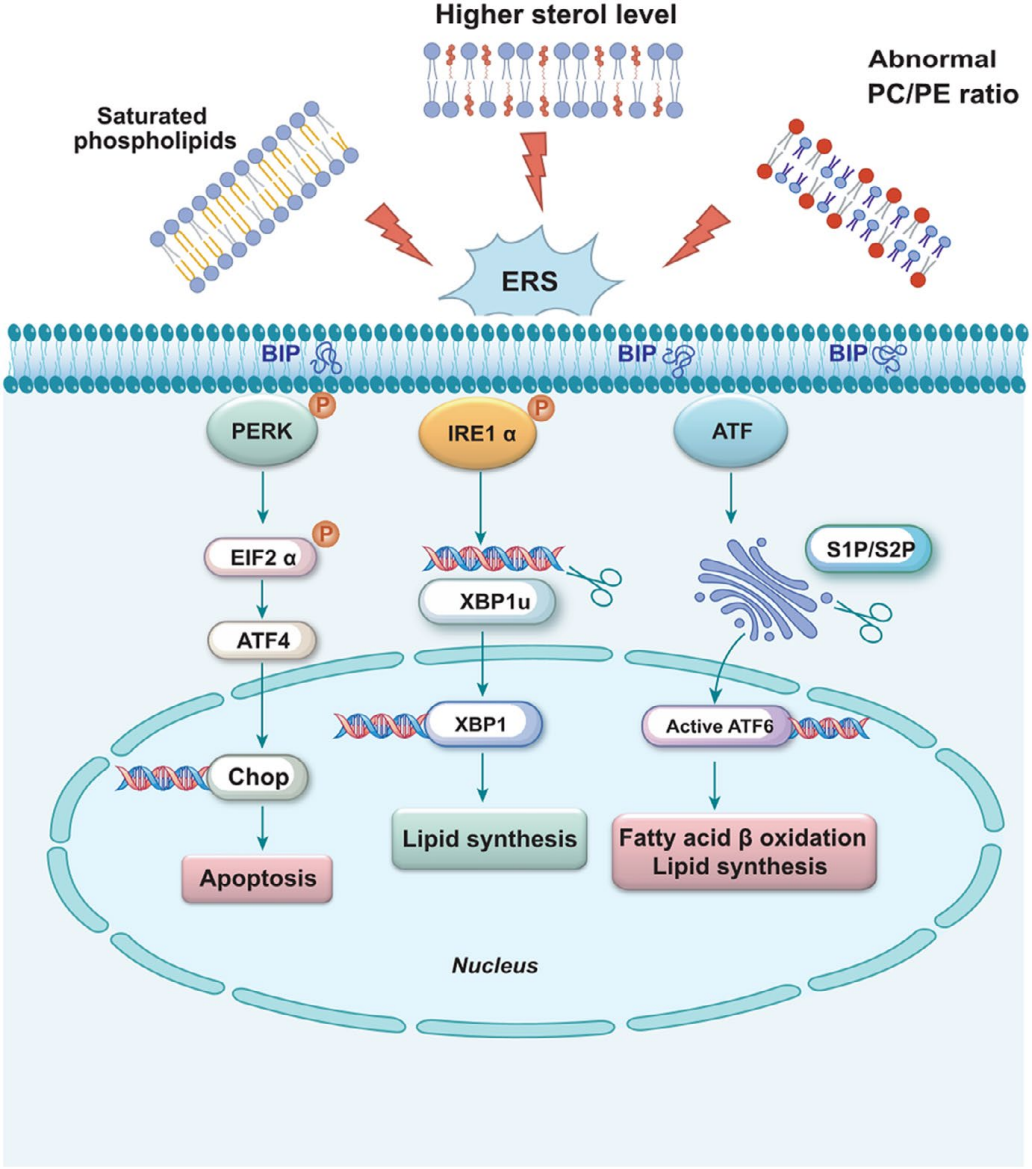
The accumulation of various lipid species in the ER membrane triggers ERS and subsequently activates the UPR. For instance, saturated phospholipids promote membrane rigidity, elevated levels of sterols are observed, and abnormal PC/PE ratios arise. BiP/GRP78 dissociates from PERK, IRE1, and ATF6 to bind to an unfolded or misfolded protein for sensor activation.

There is increasing evidence supporting the association between various physiological and pathological conditions, such as obesity, diabetes, neurodegenerative diseases, and cancer, with the accumulation of misfolded proteins within the endoplasmic reticulum, thereby triggering ERS. Prominent indicators of the ERS response, including glucose‐regulated protein 78 (GRP78) and X‐box binding protein 1 (XBP‐1), are widely utilized as biochemical markers for clinical monitoring of ERS [41]. As shown in Figure 2, IRE1α autophosphorylation induces XBP1‐mRNA specifically regulating the expression of genes involved in lipid synthesis. PERK is activated through phosphorylation of eukaryotic translation initiation factor 2α (p‐eIF2α) selectively enhancing transcription of the apoptotic chop gene. Following processing by proteases S1P and S2P, ATF6 translocates to the Golgi apparatus before cleaved ATF6 translocates to the nucleus, inducing fatty acid β oxidation and promoting gene expression related to lipid synthesis. Genetic and environmental factors such as viral infections, alterations in calcium levels, and changes in lipid content and composition can disrupt ER homeostasis. Furthermore, protein misfolding, along with dysregulation of lipid metabolism, has been implicated in a range of neurodegenerative diseases, while aberrant lipid regulation may contribute to central nervous system disorders or behavioral deficits [42, 43, 44].

The activation of ERS pathways has been found to potentially induce abnormal lipid metabolism and trigger inflammation [45]. The conformation and modification of lipid macromolecules are typically associated with changes in the structure and functionality of lipid bilayers, which encompass various types of lipids, such as fatty acyl groups, glycerides, glycerophospholipids, sphingolipids, etc. [46] Fatty acids produce three types of esters: triglycerides, phospholipids, and cholesterol esters. Phospholipids are responsible for the formation of neuronal membranes and the regulation of membrane proteins such as receptors, enzymes, and ion channels; they also serve as precursors for secondary messengers involved in inflammatory signaling. Moreover, phospholipids are indispensable for maintaining the structural integrity of neurons and promoting neuronal plasticity [47, 48].

#### 3.1.1 Phospholipids

The ER is vital in lipid biosynthesis, particularly crucial membrane lipids such as phosphatidylcholine (PC) and phosphatidylethanolamine (PE). Maintaining lipid homeostasis within the ER is closely associated with the regulation of the unfolded protein response (UPR) [49, 50]. Phospholipids predominantly constitute the membranes of mitochondria and the ER, where PC and PE regulate membrane integrity. Perturbations in PC and PE levels ultimately induce stress on the lipid bilayer, leading to ER stress (Figure 2) [51]. Low ratios of PC/PE have been linked to non‐alcoholic fatty liver disease, type 2 diabetes, cardiac disorders, and muscular dystrophy [52, 53]. Mammalian cells possess two nascent biosynthetic pathways for PC production. The cytidine diphosphate‐choline pathway, discovered in the 1950s, is primarily responsible for tissue‐specific synthesis of PC; meanwhile, PEN‐methyltransferase (PEMT) catalyzes the continuous methylation of the amino head group of PE to synthesize PC [54, 55]. Exogenous intake of PC has been shown to inhibit proinflammatory transcription in TNF‐α treated Caco2 cells. In addition, the low concentration of the major membrane lipid PC in gastrointestinal mucous membranes observed in patients with ulcerative colitis suggests that defects in colon PC metabolism may be involved in its pathogenesis and progression [56]. Aberrant lipid metabolism is implicated in the pathophysiology of AD. Whiley et al. investigated plasma lipids in AD using a multiplatform screening approach and identified three PC molecules—PC 16:0/20:5, PC 16:0/22:6, and PC 18:0/22:6—that were significantly diminished in AD cases [57]. Phosphatidylserine (PS), which constitutes 13%–15% of phospholipids in the human cerebral cortex, is a major acidic phospholipid class. As an essential component of protein docking sites in signaling pathways, PS interacts with Akt and protein kinase C to facilitate the growth and differentiation of nerve cells [58]. Studies involving PS supplementation in rodents have demonstrated improvements in memory, learning capacity, and other cognitive parameters [59].

#### 3.1.2 Fatty Acids

Differences in lipid composition and fatty acid structure can impact ER homeostasis, potentially disrupting the Toll‐like receptor 4 (TLR4) pathway when saturated fatty acids are replaced by Ω‐3 PUFA. This substitution may lead to a reduction in pro‐inflammatory markers in the aorta, including Interleukin‐1β (IL‐1β), TNF‐α, phosphorylated nuclear factor kappa B (pIκBα), phosphorylated κB inhibits protein kinase β (pIKKβ), as well as unfolded protein response markers ATF6 and GRP78 [60]. As essential components of brain nerve cell lipids and potent anti‐inflammatory bioactivators, Docosahexaenoic acid (DHA) and Eicosapentaenoic acid (EPA) are crucial for maintaining cognitive function and protecting against biological damage. PC composed of various fatty acids can mitigate cognitive decline and provide neuroprotective effects, with EPA and DHA contributing significantly to these benefits [61]. DHA influences neuronal development and neuroprotection in the formation of PC, PE, and PS [62]. Numerous studies have demonstrated that Ω‐3 PUFA, including DHA, possess anti‐inflammatory and immunomodulatory properties and show promise in the prevention or treatment of chronic inflammatory conditions such as inflammatory bowel disease, rheumatoid arthritis, asthma, and neurodegenerative diseases [63, 64]. Tan et al. reported that both exogenous and endogenous DHA can protect neurons from Aβoligomer induced damage in the pathogenesis of AD by reducing ERS and preventing cell apoptosis [65]. Therefore, it can be concluded that DHA exhibits neuroprotective effects by promoting cerebral cell growth and enhancing memory function.

#### 3.1.3 Cholesterol

Cholesterol, the most abundant sterol, regulates membrane fluidity and permeability, as well as neuronal plasticity and synaptic remodeling [66]. Among all tissues in the body, the central nervous system contains the highest concentration of cholesterol, with its synthesis and circulation being crucial for maintaining normal neurophysiology. Conversely, aberrant cholesterol metabolism in the brain has been implicated in various neurodegenerative disorders [67]. Cholesterol, predominantly present in neuronal membranes and lipid rafts, is highly susceptible to oxidation [68]. The resultant oxidized derivatives of cholesterol significantly influence cellular function, with their synthesis primarily occurring within the ER. Disturbances in cholesterol homeostasis disrupt proper ER functioning and trigger abnormal activation of the UPR (Figure 2) [49]. Lipid rafts, characterized by their high content of cholesterol, are particularly susceptible to oxidative stress and modulate ER protein activity as well as protein folding or transport through localized alterations in membrane structure.

It is widely acknowledged that ERS disrupt the regulation of lipid metabolism and lipogenesis genes, while there is a bidirectional relationship between lipid metabolism and ERS [69]. However, the precise underlying mechanism remains elusive. Mechanistically, the accumulation of free cholesterol in the lumen of the endoplasmic reticulum induces ERS [70]. Excessive retention of free cholesterol in macrophages leads to cholesterol accumulation, thereby triggering the UPR, which occurs at various stages of atherosclerotic lesions [71]. Furthermore, animal studies have demonstrated that decreasing cholesterol levels is associated with a reduction in amyloid deposition in the central nervous system, while increasing dietary cholesterol intake promotes the accumulation of amyloid. In a sample of patients with AD, elevated cholesterol levels were found to be correlated with lower Mini‐mental state examination scores compared to normal individuals [72].

As cholesterol‐lowering medications, statins have been proposed as potential therapeutic agents for neurodegenerative disorders. Salari evaluated the neuroprotective effects of simvastatin against trimethyltin (TMT) induced memory decline and hippocampal neurodegeneration. In TMT‐treated animals, simvastatin administration resulted in reduced learning and memory deficits in behavioral tasks, decreased acetylcholinesterase activity, and diminished Alzheimer's pathology markers such as presenilin‐1 and hyperphosphorylated Tau. Additionally, simvastatin treatment reduced astrocytic and microglial reactivity in the TMT group [73]. The misfolding of Aβ is a key feature of AD. Gao demonstrated that Aβ ion channels facilitated Ca^2+^ influx in the presence of 15% cholesterol but inhibited this influx at higher cholesterol levels. Consequently, excessive cholesterol may disrupt the regular distribution of the lipid bilayer, preventing ion flux through the membrane [74].

### 3.2 Lipids Affect SERCA2b Activity and Mediate ERS

The accumulation of diverse lipid species in the ER membrane triggers ERS, subsequently activating the UPR. For example, saturated phospholipids promote membrane rigidity, elevated levels of sterols are observed, and abnormal PC/PE ratios arise (Figure 2). The functionality of membrane proteins is influenced by factors such as lipid bilayer thickness and specific phospholipid composition [75, 76, 77]. The high concentration of Ca^2+^ within the ER influnce protein folding and maturation. The primary function of the SERCA pump is to transport Ca^2+^ from the cytoplasm back into the ER. Therefore, activated SERCA regulates Ca^2+^ levels within the endoplasmic membrane, thereby preserving ER integrity [78]. Among the ten different subtypes identified in humans, SERCA2 is predominantly expressed in the central nervous system [79]. Alternative splicing of SERCA2 mRNA generates two main isoforms: SERCA2a and SERCA2b. Notably, SERCA2b is ubiquitously expressed across various cell types, including neurons, cardiac muscle fibers, and smooth muscle cells [79, 80]. Baba‐Aissa et al. demonstrated through immunoblotting and in situ hybridization that the highest levels of SERCA2b expression are found in hippocampal pyramidal cells and the cerebral cortex [81]. As the sole SERCA subtype expressed in astrocytes, SERCA2b is ubiquitously present in neurons throughout the brain. In contrast, SERCA2a exhibits weak expression in the brain and is predominantly localized to layers II‐V of the cerebellum [82]. The “Calcium hypothesis of AD with aging” has been proposed to elucidate the cellular mechanisms underlying neurodegeneration and cognitive decline associated with aging [83]. The SERCA pump plays a crucial role in maintaining cellular Ca^2+^ homeostasis by actively transporting Ca^2+^ ions back into the lumen of the ER, thereby preserving low cytosolic calcium levels [84]. Conversely, decreased ER Ca^2+^ levels and elevated cytoplasmic Ca^2+^ levels are linked to ERS induced apoptosis and neurodegeneration, which contribute to Aβ accumulation and hyperphosphorylation of tau proteins [85]. Given the constitutive expression of SERCA2b, this isoform is anticipated to play a crucial role in Ca^2+^ extrusion from the cytoplasm in neuronal cells due to its high affinity for Ca^2+^ [86]. The significance of SERCA pumps in ERS is highlighted by thapsigargin, a widely used pharmacological ER stressor that specifically inhibits SERCA2b activity [87]. Furthermore, the inhibition of SERCA2b caused by the enrichment of free cholesterol or 14:0–18:0 PC in the ER membrane demonstrates a strong correlation between increased membrane lipid order and reduced SERCA2b activity [88]. Based on previous studies, chronic exposure to saturated fatty acids (SFAs), particularly palmitic acid, induces significant G2/M phase cell cycle arrest in human neuroepithelioma cells. This effect is associated with elevated ERS marked by increased p‐eIF2α and accelerated accumulation of Aβ [89]. Importantly, palmitate levels are elevated in the cerebrospinal fluid of obese individuals, and neurotoxic astrocytes have been shown to induce neuronal cell death via saturated lipids, suggesting that direct exposure to SFAs may lead to neurodegeneration by targeting the endoplasmic reticulum [90]. In contrast, monounsaturated fatty acids, such as oleic acid, completely inhibit palmitic acid mediated neuroinflammation, ERS, and proopiomelanocortin upregulation [91]. Griffin et al. suggested a potential role for SERCA2 in the development of cognitive symptoms in palmitate treated hypothalamic neurons. Exposure of neuronal Neuro2A cells to elevated palmitate levels induced several ERS features, including the activation of XBP1s and ATF6, as well as increased abundance of GRP78/Bip and SERCA2, as detected by immunofluorescence microscopy. Consequently, neurons may compensated for SFAs induced reductions in SERCA2 activity and the associated ER stress by upregulating SERCA2 expression [92].

#### 3.2.1 The Lipid Chain Structure and the Thickness Affect Membrane Fluidity

**FIGURE 3:**
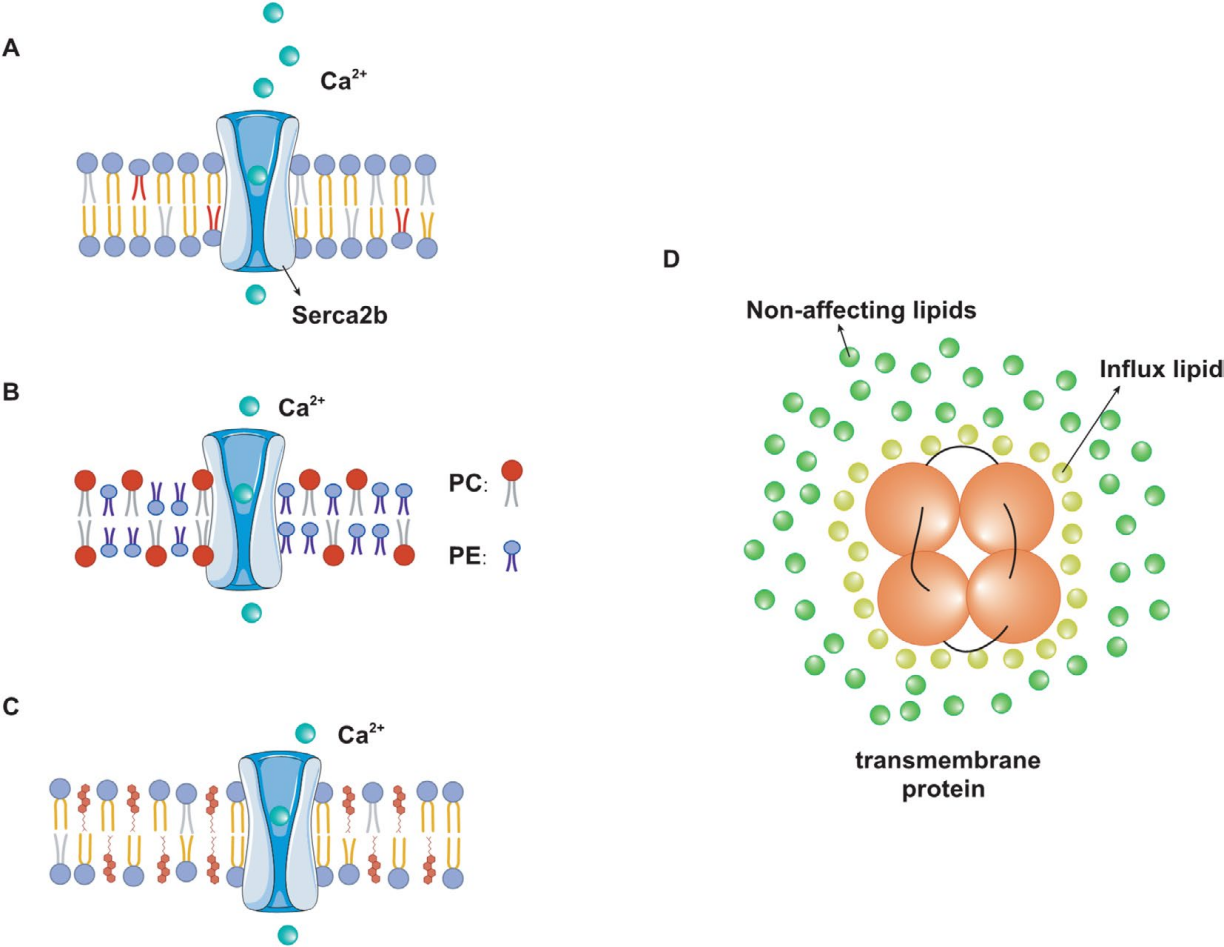
The lipid environment regulates the structure and activity of proteins. (A) The high content of saturated fatty acids in phospholipids enhances membrane order, attenuates Serca2b activity, and induces the efflux of calcium ions from the ER to the cytoplasm. (B and C) A low PC/PE ratio and elevated sterol levels reduce membrane fluidity while altering Serca2b activity. (D) An annular lipid shell forms stable interactions with transmembrane protein domains (TMDs).

The lipid chain structure and the thickness of the lipid bilayer are important in determining membrane fluidity (Figure 3) [93]. Molecular dynamics simulations revealed that the oligomerization of SERCA is influenced by the thickness of the lipid bilayer. Cholesterol or 14:0–18:0 PC in ER enhances the lipid order within the membrane. SERCA2b, as a conformationally active protein, loses its activity due to reduced conformational freedom in the cholesterol‐ordered membrane [94]. Membrane thickness, head group composition, and lipid saturation directly impact the maximum flow rate and Ca^2+^ affinity of the SERCA pump. The proper functioning of SERCA relies on the fluidity of liquid crystalline bilayers, with higher activity observed in 18:1 PC compared to 18:0 PC [95]. The maximum activity (Vmax) of SERCA depends on this bilayer thickness and can adapt through subtle conformational changes. The presence of anionic lipids PS and phosphatidylinositol phosphoric acid in lower concentrations in the endoplasmic membrane increased Vmax of SERCA [96]. Analysis of the SERCA‐Phospholamban (PLB) system reveals that phosphorylation of PLB, an endogenous regulator, induces lipid disordering, attenuates SERCA inhibition, and modulates the charge of lipid head groups to control the balance between order and disorder in SR membrane [97].

#### 3.2.2 Nannular or Nonannular Lipid Interacts With SERCA2b Protein, and Affects the Activity of SERCA2b

Proteins are adapted to a high membrane fluidity environment of lipid bilayer with the presence of an annular lipid shell, consisting of lipid molecules bound tightly to the surface of integral membrane proteins. Marsh utilized the electron spin resonance technique to determine the quantity of lipid molecules binding to the SERCA2b protein, and their number was found to be consistent with the perimeter of the protein's transmembrane region. This observation provides evidence for the existence of a distinct lipid molecular shell resembling a ring surrounding the SERCA2b protein [98].

The regulation of ion channel activity by specific lipid molecules, such as the modulation of K^+^ channels by PIP2, is widely recognized as an essential component of cellular electrical signaling [99]. Arginine residues utilize phospholipids as anchoring points for conformational changes, thereby revealing the presence of a non‐cyclic lipid binding site located within the luminal regions of transmembrane helices M2 and M4 in ER/mitochondrial membrane‐cytoplasmic lobules [78]. The research provides evidence that the SERCA2b protein is surrounded by both annular and non‐annular lipids, which interact with the SERCA2b protein (Figure 3). Additionally, it demonstrates that the unsaturation and acyl chain length of lipids have an impact on the activity of SERCA2b.

### 3.3 ERS Mediates the Expression of Gut‐Brain Axis Inflammation

The major bacterial species in the intestinal tract, such as the abundant gram‐negative bacteria *Bacteroides fragilis* and *Escherichia coli* , secrete pro‐inflammatory neurotoxins that significantly disrupt neuronal homeostasis in the central nervous system [100, 101]. Brain lysates from the hippocampus and superior temporal neocortex of patients with AD exhibited higher levels of bacterial LPS compared to age‐matched controls. The average LPS levels increased by two‐fold in the neocortex and three‐fold in the hippocampus, while advanced AD cases showed a remarkable 26‐fold increase in LPS concentration. These findings suggest that LPS derived from the intestinal microbiome promotes inflammatory degeneration within the central nervous system [5, 102].

**FIGURE 4:**
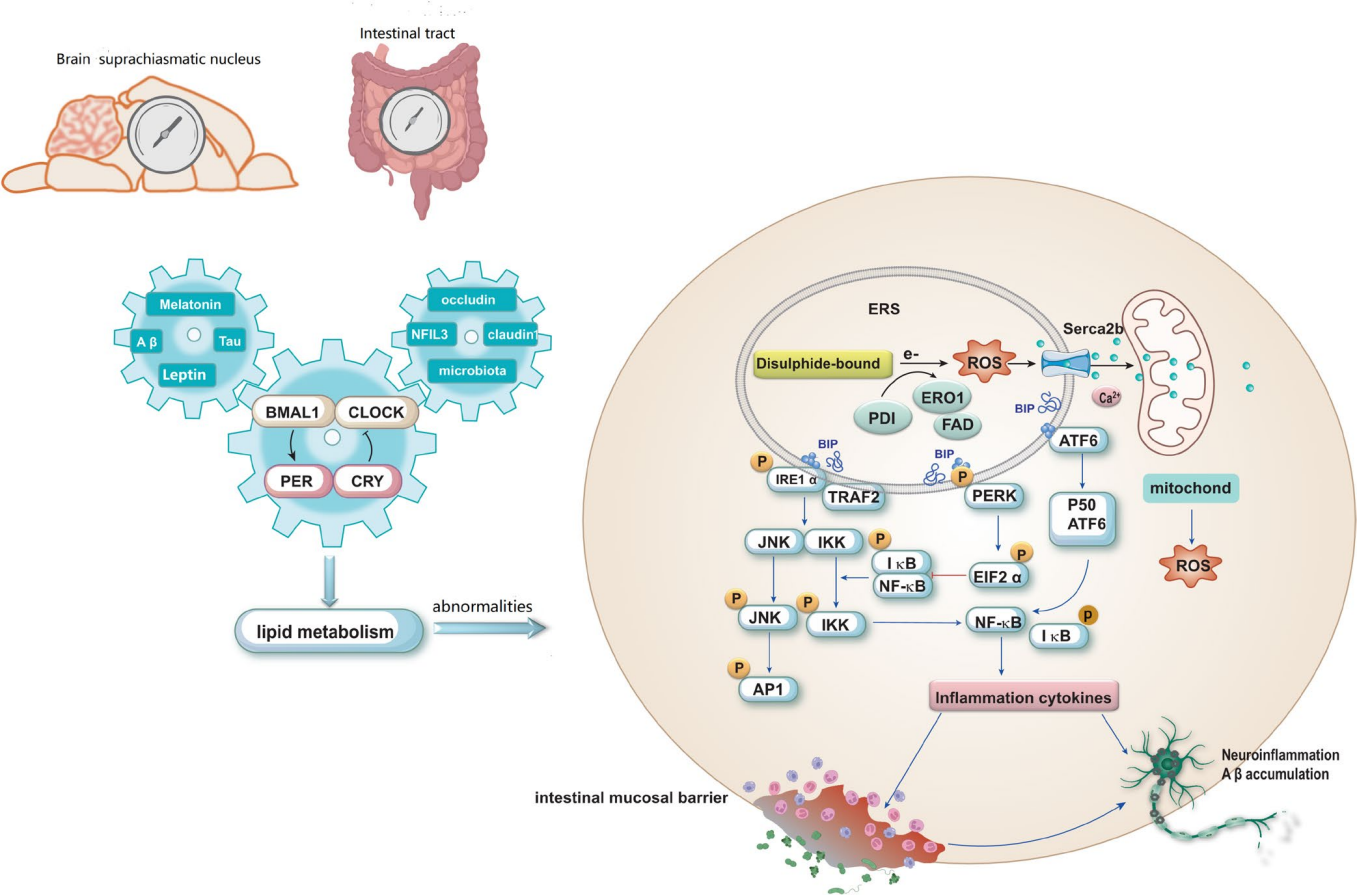
The gut‐brain axis circadian rhythm gene, CLOCK‐BMAL1 heterodimer, regulates the PER and CRY genes. The circadian rhythm disruption resulted in lipid metabolism disorders and the induction of ERS. Protein folding is an oxidative process generating reactive oxygen species (ROS). During this process, PDI accepts electrons from the protein folding substrate and oxidizes the mercaptan group in cysteine residues of proteins to form disulfide bonds. ERO1 utilizes the REDOX active coenzyme FAD to transfer electrons from PDI to molecular oxygen (O_2_), thereby generating ROS. Subsequently, ROS selectively targets Ca^2+^ channels within the ER, leading to Ca^2+^ release into the cytoplasm and triggering an ERS response. The released Ca^2+^ accumulates in the mitochondrial matrix, disrupting the function of the electron transport chain and promoting further production of ROS. Disruption of ER Ca^2+^ homeostasis perturbs the process of protein folding, leading to ERS and triggering UPR.

Therefore, maintaining an intact intestinal mucosal barrier is crucial for preventing the translocation of LPS into other tissues and organs. Activation of NF‐κB is closely associated with ERS. Phosphorylation of IRE1α's cytoplasmic domain activates TNF‐α receptor‐associated factor 2 (TRAF2), subsequently activating c‐Jun N‐terminal kinase (JNK) or κB kinase (IKK). Activated JNK then phosphorylates transcription factor activating protein 1(AP1). The activation of IKK triggers the degradation of IκB and subsequent nuclear translocation of NF‐κB, thereby promoting its activity. PERK‐mediated translational attenuation modulates the ratio between IκB and NF‐κB, facilitating the release of NF‐κB for nuclear translocation. ATF6 (p50) undergoes translocation to the Golgi apparatus, where it is cleaved by S1P and S2P proteases to generate active fragments. Active ATF6 (p50) then translocates to the nucleus and regulates gene expression associated with ERS (Figure 4) [103]. The interplay between UPR signaling pathways and lipogenesis, as well as the interaction between the accumulation of inflammatory factors and the induction of UPR, contributes to systemic chronic inflammation.

#### 3.3.1 ERS Mediates Intestinal Inflammation

Excessive ERS is a characteristic feature of enteritis pathogenesis, which can lead to the apoptosis of intestinal epithelial cells and compromise epithelial integrity. Previous studies have demonstrated the effectiveness of probiotics in ameliorating intestinal inflammation. Notably, *prevotella histicola* , an emerging probiotic strain, exhibits significant anti‐inflammatory activity by inhibiting the IRE1α‐JNK and NF‐κB signaling pathways to alleviate chronic colitis induced by sodium glucan sulfate [104]. Furthermore, mannose facilitates the normalization of protein N–glycosylation and enhances the binding of glyceraldehyde phosphate dehydrogenase to TNF‐α mRNA, while directly reducing glyceraldehyde 3‐phosphate levels to inhibit TNF‐α production in macrophages and reduce such stress [105]. PC, primarily synthesized through the cytidine diphosphate‐choline pathway in mammalian tissues, prevents ERS across diverse cell types. Studies utilizing mouse models with impaired intestinal PC synthesis have demonstrated that reduced colon PC concentration leads to adult‐onset colitis [106, 107].

#### 3.3.2 ERS Mediates Brain Inflammation

Neuronal ERS has been demonstrated to exhibit a positive correlation with microglial activation, thereby promoting the inflammatory microenvironment in neurodegenerative diseases and leading to neurotoxicity (Figure 4). Recombinant NLR Family, Pyrin Domain Containing Protein 3 (NLRP3), an inflammasome present in the central nervous system, can be activated by Aβ protein. The initial initiation signal induced by the toll‐like receptor/nuclear factor NF‐κB pathway enhances the expression of IL‐1β and NLRP3, thus promoting inflammasome formation [108, 109]. Aβ oligomer is considered a ligand for TNF‐α receptor and TLR4 [110].

Numerous studies have demonstrated that Aβ oligomers can increase apoptosis by activating the TNF‐α pathway and inducing ERS [111]. Chronic dysfunction of the ER is strongly associated with impairment in memory and cognition, while downregulation of PERK expression prevents aberrant phosphorylation of eIF2α (Figure 4) [112]. Perturbations in cellular Ca^2+^ homeostasis are a prominent characteristic of AD, characterized by elevated levels of Ca^2+^ observed in neurons and astrocytes within mice. Particularly, basal Ca^2+^ levels surrounding astrocyte Aβ plaques are significantly heightened [113]. Under conditions of ERS, disruptions of brain tissue's Ca^2+^ homeostasis promote abnormal accumulation of Aβ protein. Additionally, IRE1‐induced NLRP3 facilitates the aberrant aggregation of Aβ protein and the formation of neurofibrils composed of hyperphosphorylated Tau protein [114].

## 4 Dietary Lipids and Alzheimer's Disease

Optimal dietary patterns have been demonstrated to attenuate oxidative stress and inflammation, promote cardiovascular and brain health in older adults, thereby exerting preventive effects against AD (Table 1) [2, 115, 116, 117]. It is widely acknowledged that AD is associated with systemic inflammation. The inclusion of Ω‐3 fatty acids in one's diet not only influences the composition of gut microbiota, but also suppresses LPS production while reducing levels of metabolic endotoxins [118]. In addition, its lipid metabolites function as anti‐inflammatory components that can repair intestinal inflammation damage. DHA is enzymatically transformed into specialized lipid mediators, such as protectin and resolvin, which prevent neutrophil adhesion to blood vessel linings and invasion into the colon through lubrication of neutrophils [119]. Partial substitution of saturated fatty acids with flaxseed oil, rich in Ω‐3 fatty acids, has been shown to potentially inhibit cardiovascular risk markers, pro‐inflammatory cytokines, as well as aortic ERS sensors and effectors [60]. Administration of EPA‐PC or DHA‐PC has demonstrated the ability to reduce NLRP3 levels in AD mice [120]. EPA and DHA are essential Ω‐3 fatty acids that cannot be synthesized by the human body and must be obtained from the diet. Therefore, selecting a dietary oil abundant in Ω‐3 fatty acids is important to protect the integrity of the intestinal mucosal barrier and prevent AD. Fish oil and antarctic krill serve as a plentiful reservoir of Ω‐3 PUFAs. Studies have reported that n‐3 PUFA‐PLs from squid seeds ameliorate cognitive deficits in Aβ induced AD rat models, while long‐term administration of pure EPA ester decreases memory impairment induced by IL‐1β administration [121]. Modulating the dietary ω‐6/ω‐3 fatty acid ratio can influence Aβ production and age spot formation in the hippocampus of mice [122].

**TABLE 1:** Effects of lipid diets on animal models of AD.

Dietary intervention lipids	Animal model	Major finding
HF+ flax seed oil (10% of total caloric value)	Male Swiss and LDLr‐KO mice	The ω3 fatty acids contribute to the inhibition of cardiovascular risk markers, pro‐inflammatory cytokines, and ER stress sensors and effectors in the aorta [60].
EPA‐PC, EPA‐EE, and DHA‐EE (DHA + EPA = 60 mg/kg·d)	Aβ1‐42‐induce AD rats	The neurotoxicity induced by Aβ1‐42 could be inhibited by EPA‐PC through mitigating the activation of NLRP3 inflammasome and enhancing autophagy [120].
Coconut and soybean oil enriched with ethyl‐EPA	Wistar rats injected with IL‐1β	E‐EPA attenuates the levels of the inflammatory mediator prostaglandin E2 in the hippocampus [121].
ω‐6/ω‐3 ratio of 24.6%; and Lower ratio of 3.2%	Double‐transgenic APP/PS1 mice	The higher the ω‐6/ω‐3 ratio, the lower the levels of ceramides and the higher the levels of FAs, specifically docosatetraenoic acid [122].
EPA‐PL group 150 mg/kg/day, 300 mg/kg/day	Aβ1–40‐induced cognitive deficiency	The EPA‐PLs effectively mitigated Aβ—induced neuroto‐ xicity, encompassing oxidative stress, apoptosis, the neuroinflammatory cascade, and hyperphosphorylated tau [123].
High fat diet	Male fat‐1 transgenic mice	HFD‐fed fat‐1 mice exhibited preserved mucus layer integrity and a mitigated decline in mucin expression mediated by ER stress [124].
Supplementation by Ω‐3.	Alzheimer's disease patients	The Ω‐3 treatments exhibited an upregulation of energy enzymes involved in glycolysis and the citric acid cycle, along with the anti‐inflammatory circadiangenes CLOCK and ARNTL2 [125].
DHA administration	Male fat‐1 transgenic mice	Administration of DHA could potentially confer neuroprotection against Aβ oligomer‐induced injury by attenuating ERS and inhibiting cellular apoptosis [126].
DHA‐PC and phosphatidylserine (DHA‐PS)	SAMP8 AD model mice	Administration of DHA‐PC and DHA‐PS significantly restored lipid homeostasis in a murine model of dementia [127].
Fish oil, and a HFD supplemented with algae oil.	Male C57BL/6 mice	Fish oil and algae oil mitigate circadian dysregulation of gut microbiota in high‐fat diet‐induced mice [128].

Dietary fatty acids, serving as a crucial structural component of the cell membrane, are associated with both pro‐inflammatory and anti‐inflammatory mediators. Maintaining a balance in the quantity and proportion of lipids consumed is important for overall well‐being. Studies have focused on regulating circadian rhythm to improve liver circadian clock function and lipid homeostasis in high‐fat mice [129]. Li reported a correlation between the regulation of clock gene expression and the attenuating effect of oleic acid‐induced lipid accumulation in HepG2 cells [130]. Circadian rhythms exert an influence on the bidirectional communication between the intestinal tract and central nervous system [131]. Chen et al. documented an elevated risk of AD among patients with irritable bowel syndrome [132]. In conclusion, favorable dietary habits can modulate neurophysiological function to optimize health.

## 5 Conclusion

**FIGURE 5:**
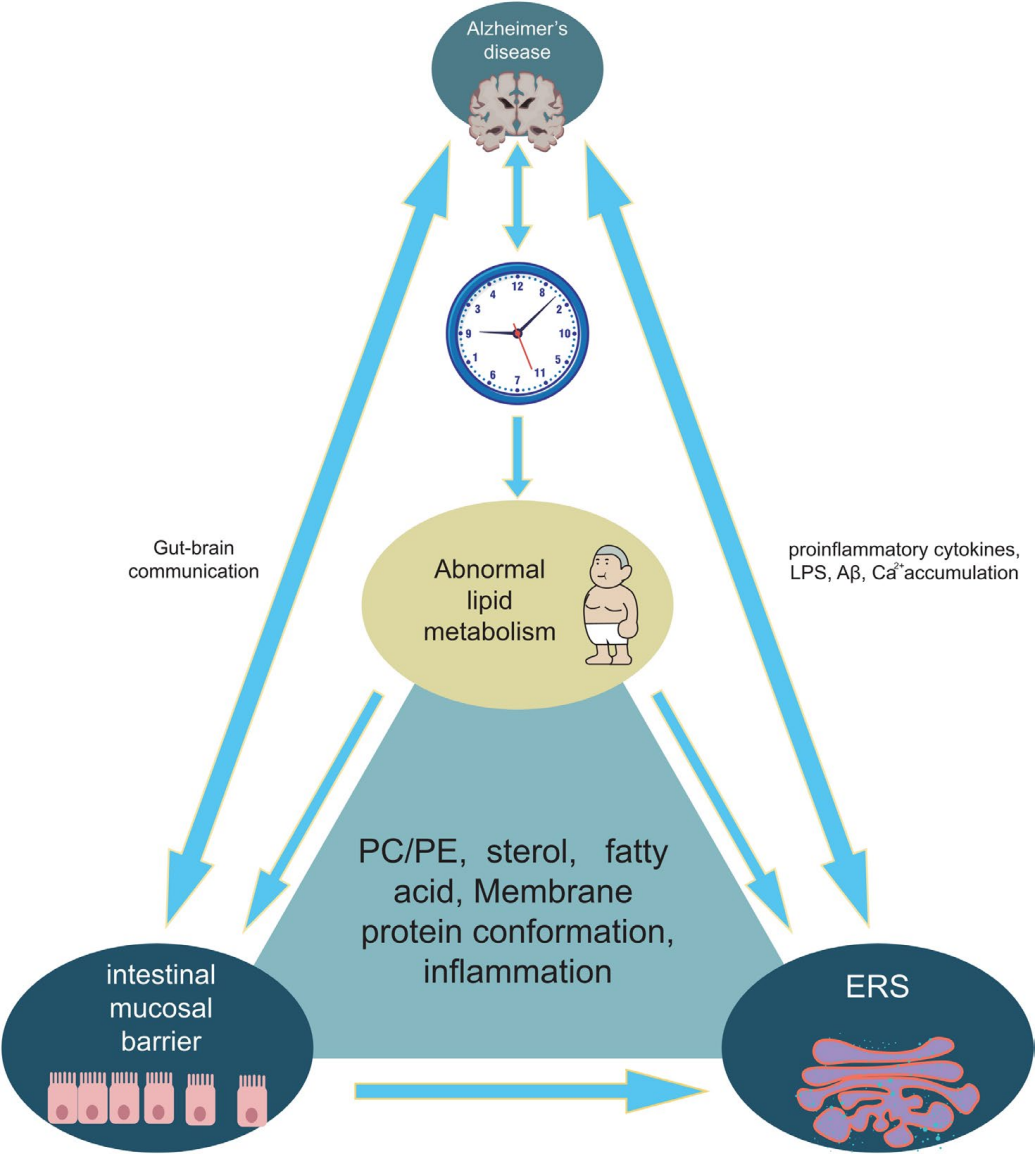
Circadian rhythms regulate lipid metabolism, thereby influencing the lipid composition of ER and inducing conformational changes in the transmembrane protein SERCA. Specific lipid components interact with SERCA to modulate its activity and trigger the ERS response. Intestinal mucosal ERS leads to increased permeability and accelerates AD progression.

In summary, this article presents a comprehensive review of the mechanisms underlying lipid metabolism in AD (Figure 5). Emerging evidence from animal models and clinical trials suggests a complex bidirectional relationship between AD and circadian rhythm, as illustrated in Figure 1. Significant variations in lipid composition across different microdomains impact protein conformation; thus, optimizing ER lipid composition represents an effective approach for inhibiting UPR. This review emphasizes the associations among circadian rhythm, lipid homeostasis, AD, and diet. Modulating dysfunctional UPR through lipid metabolism offers greater potential for new strategies that focus on inflammatory components.

## Author Contributions

Jianhui Su and Zhe Tang contributed to the study concept; Lanyang Zhao and Runze Fu designed the structure of the manuscript; Jianhui su, Runze Fu, and Zhe Tang collected academic paper materials; Lanyang Zhao and Runze Fu performed the drafting of the article; and Jiahui Su and Zhe Tang revised the article for intellectual content and provided financial support.

## Conflicts of Interest

The authors declare no conflicts of interest.

## Data Availability

In this review, we searched PubMed, Web of Science, and Elsevier SD up to 2024 for relevant studies. PubMed: https://pubmed.ncbi.nlm.nih.gov/, Web of science: https://www.webofscience.com/, Elsevier SD: https://www.sciencedirect.com/.
